# Finding Yaws among Indigenous People: Lessons from Case Detection Surveys in Luzon and Visayas Island Groups of the Philippines

**DOI:** 10.4269/ajtmh.22-0566

**Published:** 2022-12-26

**Authors:** Belen Dofitas, Maria Christina Batac, Jan Hendrik Richardus

**Affiliations:** ^1^ Department of Dermatology, College of Medicine, University of the Philippines Manila, Manila, Philippines;; ^2^ Department of Public Health, Erasmus MC, University Medical Center Rotterdam, Rotterdam, The Netherlands

## Abstract

Yaws is a chronic, highly contagious skin and bone infection caused by *Treponema pallidum* subspecies *pertenue*, usually affecting children in impoverished and remote communities. Yaws lesions have thick yellow crusts on pink papillomas that ulcerate and leave deep scars. Yaws cases were confirmed in the Liguasan Marsh, Mindanao Island group, Southern Philippines, in 2017, but there were no cases confirmed in the Luzon and Visayas Island groups. We aimed to detect at least one active or latent yaws case in the island groups of Luzon and Visayas. Active yaws surveillance was conducted by inviting healthcare providers to report yaws suspects. Five remote villages were included in the case detection surveys: three in Luzon and two in the Visayas Island groups. Two indigenous peoples communities were included: Aetas of Quezon and Dumagat/Remontados of Rizal provinces. Trained field personnel conducted free skin check-ups of children, household contacts, and community members. Yaws suspects underwent point-of-care serologic tests for *T. pallidum* and nontreponemal antibodies. A total of 239 participants were screened for skin diseases, and 103 had serologic tests. Only the Aetas of Quezon province, Luzon, had confirmed yaws cases. Nineteen cases (54.3%) were detected among 35 Aetas: five active yaws (four children, one adult), two latent yaws (adults), and 12 past yaws (1 child, 11 adults). An 8-year-old boy had yaws with skeletal deformities. We report the first yaws cases among the Aetas of Quezon, Luzon Island group. Active yaws surveillance and case detection in remote areas and among indigenous peoples should continue.

## INTRODUCTION

Yaws is a chronic, contagious, nonvenereal, treponemal infection that manifests mainly in human skin as verrucous, raspberry-like nodules or as plaques with moist, yellowish surfaces or may be ulcerated occurring in children younger than 15 years.^1^ Yaws has not been officially reported in the Philippines after the last Philippine Health Statistics report in 1973. Children and adults with suspected yaws were reported by Dofitas and Kalim^2^ and Médecins Sans Frontières^3^ in the Mindanao Island group, prompting the Department of Health to commission an epidemiological study to assess the status of yaws in the Philippines. In 2017, yaws was confirmed clinically and serologically in the Liguasan Marsh area of the Mindanao Island group, making the Philippines the 14th country to be endemic for yaws. The low prevalence (0.2%) among children below 15 years of age was probably due to limited sampling.^4^

The status of yaws in the other island groups of Luzon and Visayas, however, remained unknown. A follow-up study of the first epidemiological study was deemed necessary by the Department of Health to assess the status of yaws in the rest of the country and to guide health authorities in the establishment of an appropriate yaws control and eradication program.

This report describes the search for yaws in each of the island groups of Luzon and of the Visayas in the Philippines and the discovery of the first yaws cases among the Aetas, an indigenous people in the mountains of Luzon.

## MATERIALS AND METHODS

To detect yaws cases in the major island groups of Luzon and Visayas, the Department of Health commissioned a team of researchers to search for communities that were at high risk for yaws and to conduct case detection surveys in purposively selected villages.

### Ethics.

The study proposal was approved by the Technical Review Committee of the Philippine Council for Health Research and Development and was registered in the Philippine Health Research Registry (PHRR 191212-002355). Ethical approval was granted by the Single Joint Research Ethics Board (SJREB 2018-24) and the University of the Philippines Manila Research Ethics Board (UPMREB 2018-534-01). Written informed consent to participate in the study was secured from the parents or guardians of minors and from adult participants. Written informed assent was secured from minors aged 6–17 years of age. This study involved indigenous people as participants and was conducted with the approval of the National Commission on Indigenous Peoples (NCIP).

### Active yaws surveillance to identify study sites.

Because yaws had not been a notifiable disease since 1973, we had to select sites where yaws may have been sighted historically or reported to the investigators during the study period. To identify potential study sites, we gathered active surveillance reports of yaws from private physicians (general practitioners, pediatricians, dermatologists) and government health personnel (rural health units, municipal health offices). We e-mailed information on how to recognize yaws and how to document and treat cases using the WHO Yaws Recognition Booklet for Communities^5^ and the WHO Yaws Fact Sheet^6^ to enable the health personnel to recognize yaws and report it. The health personnel were requested to answer the electronic survey inquiring about any prior encounter with yaws. We also called local health offices to invite personnel to participate in the surveillance and to send us any previous reports of yaws. Thereafter, the health personnel were requested to report and document cases of suspected yaws that may be encountered in their respective catchment areas throughout the study period.

### Study site selection.

Yaws would have higher chances of detection in high-risk communities and focal indigenous populations rather than through a random selection of villages within a municipality. Our study site selection criteria aimed to conduct the surveys in villages where yaws was more likely to exist: 1) Geographically Inaccessible and Disadvantaged Areas (GIDA) and 2) indigenous peoples.

**Figure 1.**
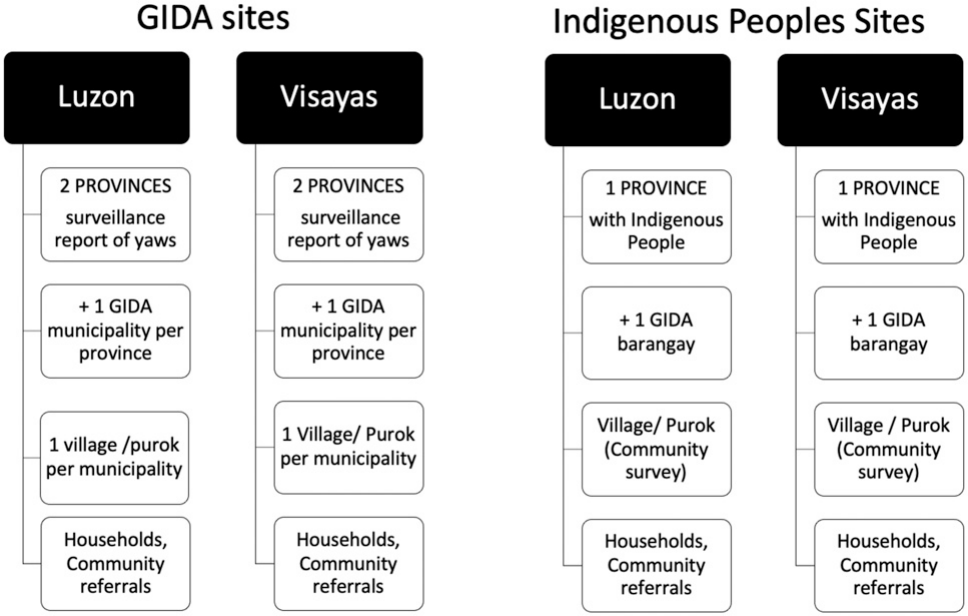
Sampling design of yaws case detection surveys in Luzon and Visayas island groups (barangay = town; purok = village). GIDA = Geographically Inaccessible and Disadvantaged Areas.

The sampling design started with the selection of a province with past or recent reports of yaws sightings, then a municipality classified as GIDA with suspected or historical presence of yaws cases. After selecting the municipalities, the investigators consulted the local health officials on the selection of villages that were most impoverished, were remote, or had indigenous peoples but were safe to conduct the study in. We also chose remote villages with indigenous people per major island group based on surveillance reports of yaws suspects from physicians. For each village, we planned a total enumeration of children 15 years old and younger (Figure 1).

### Case detection surveys: community skin screening and serological tests.

We conducted cross-sectional surveys of selected villages to screen for and detect yaws. Field teams from local health services and study coordinators were oriented and trained by the investigators using online platforms because the COVID-19 pandemic restrictions did not permit air travel or land travel of study investigators. The local health personnel discussed health information on skin diseases and yaws in public areas and through house visits because physical and social distancing was enforced throughout the pandemic. Flyers with photographs of skin diseases including leprosy and yaws were distributed as well.

For each village, all households with children 14 years old and younger were invited to receive free skin check-ups. Study health personnel performed pre-screening of the children for any pathologic skin lesions, especially lesions with appearances suggestive of yaws. Field teams comprising local health personnel conducted skin examinations of children and household contacts on scheduled free skin clinic days. We followed the recommended screening algorithm of WHO for yaws suspects and the criteria for the classification and confirmation of yaws cases: clinical skin signs suspicious of yaws combined with serological confirmation of *Treponema pallidum* antibodies and non-*T. pallidum* antibodies.^7^ All participants with skin lesions that were suspicious for yaws (i.e., yellow-crusted nodules, skin ulcers, deep atrophic scars) were considered yaws suspects. The study physicians referred these participants to the study dermatologist (BLD) for diagnosis and medical management on-site or through store-and-forward mobile phone teledermatology. WHO-recommended definitions of yaws cases are found in S1.^8^

**Figure 2.**
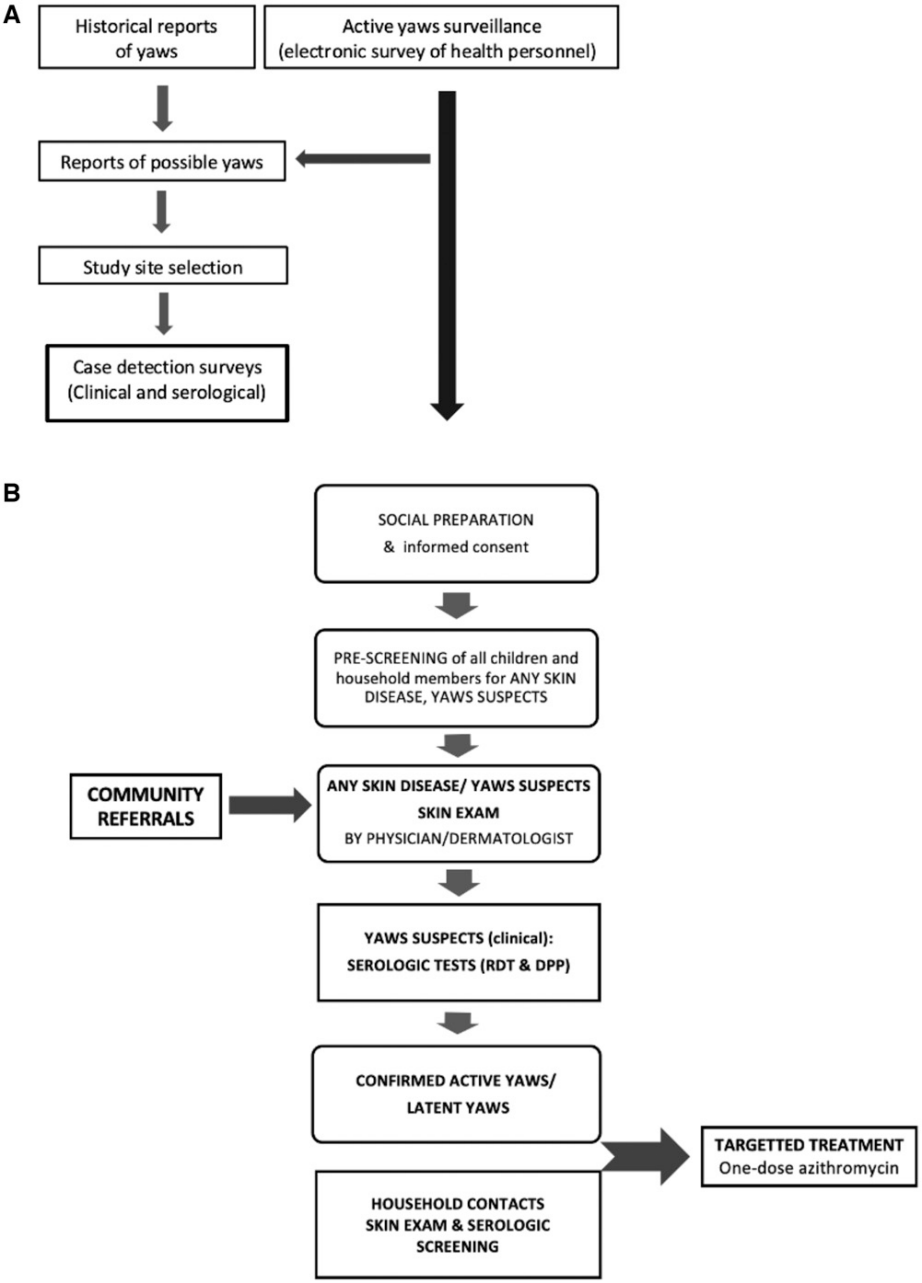
Flowchart of study procedure. (**A**) Study site selection. (**B**) Case detection.

A point-of-care test kit for treponemal antibody detection was used, SD Bioline Syphilis Rapid diagnostic test (RDT), to screen yaws suspects and their household contacts. Those with reactive treponemal antibody tests underwent a second point-of-care serologic test, the Chembio Dual Pathway Platform Syphilis Confirm and Screen (DPP), to confirm the presence of treponemal and nontreponemal antibodies. Confirmed yaws cases and household contacts were treated with one-dose azithromycin as recommended by WHO.^7^ “Rumors” about yaws from local residents and health workers were also investigated (Figure 2).

## RESULTS

### Active yaws surveillance.

The nationwide active yaws surveillance of health care personnel was conducted from August 2019 to October 2020. The total number of health personnel contacted was 3,974. Among them, 117 (2.9%) participated in the online survey. We received reports from a rural health unit doctor and a pediatrician about yaws suspects among indigenous peoples in the Luzon area: the Aetas of Tagkawayan Municipality, Quezon Province, and the Dumagat/Remontados of Rodriguez Municipality, Rizal Province. Rumors of yaws were reported in Basey, Samar province, and Negros Island, both located in the Visayas island group.

### Study sites for case detection.

Five study sites (villages) were included in the case detection surveys, including three in Luzon and two in the Visayas Island groups (S2). We could not find a third study site in the Visayas Island group due to the COVID-19 lockdowns that disrupted local government activities. The study sites were: 1) Village A, Balanga City, Bataan (Luzon); 2) Village B, Municipality of Rodriguez, Rizal (Luzon); 3) Village C, Municipality of Tagkawayan, Quezon (Luzon); 4) Village D, Municipality of Leyte, Leyte (Visayas); and 5) Village E, Municipality of Basey, Samar (Visayas).

### Participants of case detection surveys.

The five study sites had a total of 616 children, of whom 378 (61.4%) were visited by the field teams in their homes and invited to join the study. A total of 239 individuals participated in this phase of the study. Of these, 208 (87.0%) were residents of the target study sites: 160 children (66.9%) and 48 household contacts (20.1%). There were 31 community referrals (13.0%) (i.e., participants who did not reside in the target study site but wanted to be examined). The mean age of participants was 14.4 years (SD: 15.4; range: 0.17–68). There were more females (137, 57.3%) than males (102, 42.7%). A total of 103 yaws suspects (43.1% persons screened for skin disease) were tested for *T. pallidum* antibodies: 81 children, 18 household contacts, and four community referrals.

Of the five study sites, yaws cases were only detected among the Aetas, an aboriginal indigenous people, in Quezon province, Luzon island group.

### The Aeta community.

The Aetas, also known as Agta or Negritos, are an indigenous people and are regarded as one of the first inhabitants of the Philippines. They have short stature, dark brown skin, and curly hair.^9^^,^^10^ The Aetas residing in Tagkawayan, Quezon, are semi-nomadic and belong to a loose cluster of 17–19 families residing in the Sierra Madre Mountains. The number of individuals was estimated to be 59 in the year 2020. They did not have a formal leader during the time of the study. They move back and forth from the adjacent province, Camarines Norte, to Quezon province. The children went to school in Camarines Norte; therefore, the investigators could not examine the schoolchildren contacts. According to the local health officials, in 2019, the Aetas were officially registered as residents of Tagkawayan. As relatively new residents of the municipality, the Aetas had not been checked before by the local health personnel or by Department of Education personnel and had not been oriented yet by the NCIP.

Their village is a 2-hour walk up the mountain from the town center. Based on our survey, the Aetas have been panning for gold (referred to as “pagkabod”) in the river of Village C, and this has been their main source of livelihood. They spend several hours immersed in the river water. After gathering enough gold dust, they smelt it and sell the gold.

### Demographic characteristics of Aeta participants.

This study was able to screen a total of 35 out of 59 (59.3%) Aeta village members, covering 13 households, during October 2019 and September 2020. Eighteen children (30.5%) and 17 adults (28.8%) were screened. Adults who did not participate were either out of their houses to work or refused to have a skin examination. A few of the younger children refused to have the serologic test done. There were 23 male participants (69.2%) and 12 female participants (30.8%). The mean age was 19.2 years (SD: 14.4; range: 1–52 years). There were 16 children (45.8%) 14 years old and below who participated. The mean age was 7.2 years (SD: 3.8; range: 1–14), with 28.6% belonging to the 5–9 years age group.

### Demographic characteristics of yaws cases.

Thirteen out of 24 households were examined (54.2%) and had yaws cases. Among the 35 participants screened, a total of 19 (54.3%) yaws cases were detected. There were 10 male participants (58.7%) and nine females (47.4%) (mean age: 27.1 years; SD: 14.1). The majority were adults (14/19, 73.7%), and there were five participants (26.3%) in the pediatric age group (i.e., younger than 19 years old). The mean age among children who were less than 15 years old was 9 years (SD: 1.8); among the 15 years old and above age group, the mean age was 31.9 years (SD: 11.7).

There were five active, two latent, and 12 past yaws cases detected. There were four males and 1 female with active yaws. The mean age was 12.2 years (range: 7–25 years). There were two adults with latent yaws (one male, one female) with a mean age of 27 years (range: 24–30). The 12 past yaws cases had a mean age of 33.3 years (SD: 12.7; range: 16–52 years). Half of the past yaws cases (6/12) were young adults aged 19–29 years. There were six males and six females. Most of the adults (11/17, 64.7%) worked as gold panners (“pagkakabod”) (Table 1).

**Table 1:** Demographic characteristics of Aetas with yaws (*N* = 35)

	Mean age (years)			Female		Male	
Group		SD	Range	*n*	%	*n*	%
0–14 years	9	1.8	7–11	2	5.7	2	5.7
≥ 15	31.9	11.7	16–52	7	20.0	8	22.9
Total				9	25.7	10	28.6
Active	12.2	7.3	7–25	1	2.9	4	11.4
Latent	27	4.2	24–30	1	2.9	1	2.9
Past	33.3	12.7	16–52	6	17.1	6	17.1
All yaws cases	27.1	14.1	7–52	8	22.9	11	31.4

### Types of yaws detected among the Aetas.

Yaws affected more than half of the Aetas examined (54.3%). Active yaws was detected in 5/35 (14.3%) of persons screened. Among children 0–14 years old, there were four (11.4%) with active yaws. One adult had confirmed active yaws (2.9%). Latent yaws affected 5.7% of persons screened, whereas past yaws accounted for the majority of cases detected (34%) and affected adults except for one case, a 16-year-old female. The infectious cases (active and latent yaws) were 7/35 (20%).

Skeletal deformities (bowing of both forearms) were detected for the first time in one active yaws patient, an 8-year-old boy who also had yaws skin lesions. Plantar yaws were apparent in five participants (14.3%; three children, two adults). There was no case of tertiary yaws detected (Table 2).

**Table 2:** Types of yaws detected among the Aetas (*N* = 35)

Type of yaws	Children 0–14 years		≥ 15 years		Total	
	*n*	%	*n*	%	*n*	%
Active	4	11.4	1	2.9	5	14.3
Latent	–	–	2	5.7	2	5.7
Past	1	–	11	34.3	12	34.3
Tertiary	–	–	–	–	–	–
Total yaws cases	5	14.3	14	40.0	19	54.3
Periosteitis	1	2.9	–	–	1	2.9
Plantar yaws	3	8.6	2	5.7	5	14.3

### Description of yaws cases.

The details of the clinical manifestations of yaws cases are presented because this is the first report on their clinical features among Aetas in the Philippines. Clinical images are essential for the recognition of yaws in countries where there has been no surveillance. Clinical and serological details are found in S3.

#### Active yaws cases.

Five active yaws cases were detected. They were all children except for Case 5, a 25-year-old male. Cases 2, 3, and 4 were siblings. All of them had small skin ulcers, yellow-crusted nodules, black-crusted erosions and atrophic scars on the legs or dorsa of the feet. Case 1 presented with bony deformities on his arms characteristic of secondary yaws. Instead of ulcerated or crusted nodules, Case 4, an 11-year-old female, had papulosquamous plaques on the dorsa of her feet and ankles extending to the soles. Cases 2, 3, 4, and 5 had plantar yaws. Clinical photographs are in S4–S9.

##### Bony deformities due to yaws.

**Figure 3.**
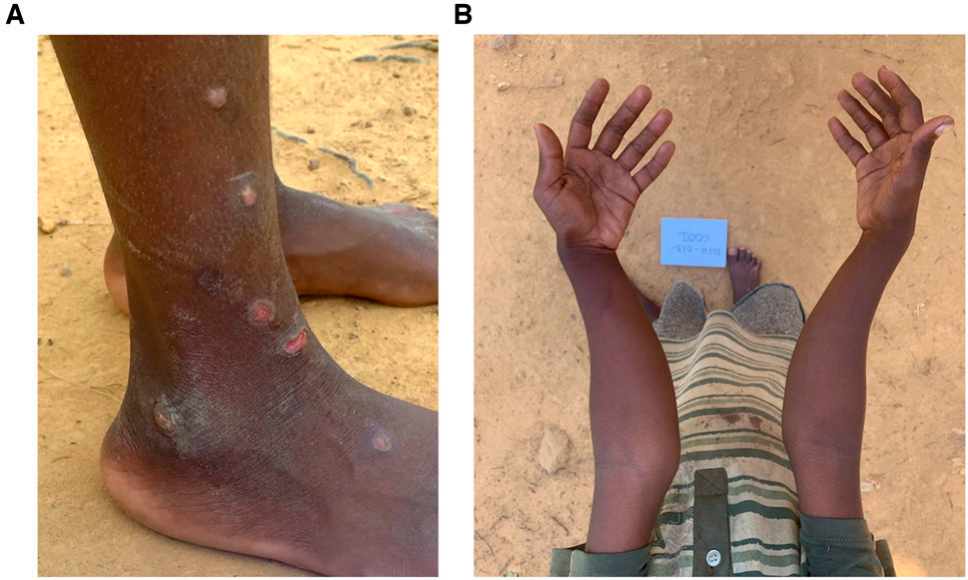
Case 1. Active yaws with bony deformities: (**A**) small ulcers and dry nodules on the leg and feet; (**B**) medial bowing of the forearms (Photo credits: Dr Kevin Matundan).

An 8-year-old male (Case 1) had small ulcers and ulcerated nodules on the dorsa of his feet and ankle area and dry nodules on the legs of 1 month duration. He reported bleeding and pain. His forearms had a bowing deformity, with the distal forearms curving in medially upon extension. His parents claim that the deformity began a few months prior to the yaws study. This was the only case of skeletal changes found among the yaws cases and the children screened for yaws (Figure 3). The DPP test confirmed the presence of both treponemal and nontreponemal antibodies. His father tested positive for *T. pallidum* antibodies only. His 7-year-old sibling, who had a large, yaws-like ulcer on the knee, tested negative for *T. pallidum* antibodies.

##### Plantar yaws.

Upon inspection of the soles of the feet, four out of five active yaws cases had plantar yaws. There were multiple healing or fresh erosions, small punched-out ulcers, or curvilinear deep wounds. Florid yaws papillomas were found in Case 5 (S10). The Aetas found yaws bothersome due to the pain of wounds on their feet as they worked in the river.

##### Histopathologic findings of yaws.

The study physician performed a punch biopsy of one skin ulcer on the ankle area of Case 1. The specimen was processed in the Philippine General Hospital and read by the dermatopathologist as “lichenoid psoriasiform dermatitis with plasma cells” consistent with yaws (S11).

#### Latent yaws.

There were two latent cases who were adults (a 30-year-old female; and a 24-year-old man) from different households. At least one other adult member of their households had past yaws. The woman also had tinea imbricata, and the male had well-defined scars on the dorsa of the feet.

#### Past yaws.

There were 12 cases with past yaws. All except one were adults. One past yaws case (a 22-year-old male) was a household contact of an active yaws case (Case 4). Three past yaws cases were household contacts of the two latent yaws cases. All had a history of recurrent papules or nodules, wounds, or ulcers on the feet and legs that were pruritic or nonpruritic. On skin examination, all of them had hypertrophic or atrophic scars on the lower extremities, especially the feet. The only child with past yaws was a 16-year-old female with a solitary, dark brown, ulcerated, and crusted nodule in the periaxillary area and multiple atrophic scars on the knees. On the dorsa of her feet, there were multiple, small, round erosions. Two patients had concomitant tinea imbricata.

### Treatment of yaws.

All participants of the Aeta community were given treatment for their skin problems and given one dose azithromycin by the rural health physician and nurse after the case detection survey. The yaws cases detected in 2019 had moved to Camarines Norte before they could be given azithromycin. The follow-up visit and serologic testing are scheduled within the next 6 months and depend on the COVID-19 problem in the municipality. The Rural Health Unit physician and nurse will continue active yaws surveillance for new migrants from the adjacent province of Camarines Norte.

## DISCUSSION

This is the second epidemiological study conducted in the Philippines to ascertain the status of yaws and to provide a basis for the type of yaws control and eradication program that the Philippine government will undertake. The first study in 2016–2017 confirmed the presence of active, latent, and past yaws in selected municipalities of the Liguasan Marsh area of Mindanao, within Cotabato, Sultan Kudarat, and Maguindanao provinces.^4^ The current study focused on the Luzon and Visayas Island groups and succeeded in confirming the presence of active, latent, and past yaws in the Luzon Island group but not in the Visayas Island group. This is also the first report of confirmed yaws cases outside the Mindanao Island group and among indigenous people in Luzon. The total number yaws cases reported after 1973 is at least 250, including the new cases detected in the current study (Table 3).^3^^,^^11^^,^^12^

**Table 3:** Summary of yaws cases (active, latent, past) reported after 1973 (Philippines)

Year	Children	> 15 years old	Total	Location	Method of detection	Reported by	References
2020	5	14	19	Luzon	Clinical and serological survey (DPP)	Dofitas B	Current study
2017	5	10	14	Mindanao	Clinical and serological survey (DPP)	Dofitas B	Dofitas et al^4^
2012	2	97 women	99	Mindanao	Serological survey (women: RDT and VDRL quanti; children RDT)	Kalim S	Kalim S 2013 (unpublished)
2009	0	25.5% pregnant women	25.5% pregnant women	Mindanao	Serological survey (prenatal screening for syphilis)	Médecins Sans Frontières	^3^
2000	77	41	118	Mindanao	Clinical survey	Dofitas B	WHO^2^

DPP = Dual Pathway Platform Assay for Syphilis; RDT = Rapid Diagnostic Test (SD Bioline Syphilis test); VDRL = Venereal Diseases Research Laboratory.

### Yaws among indigenous peoples.

The current study confirmed the presence of yaws in the Luzon Island group in one study site with indigenous people, the Aetas. When tinea imbricata cases were reported by the RHU physician in August 2019 and a skin outreach mission was conducted by this investigator (BLD), it was the first opportunity to confirm tinea imbricata and to screen the Aetas for possible yaws. Three yaws cases (one latent, two past yaws) also had tinea imbricata. The RHU physician performed the serologic confirmation of *T. pallidum* antibodies and nontreponemal antibodies using the DPP test kits in October 2019, after which the investigators began the process of securing permission from NCIP for the yaws case detection study to be conducted among the Aetas.

The overall occurrence of yaws (all types) among the Aetas of Quezon was high (54.3%) among the 35 participants examined. This is an overestimation or underestimation because around 40% of the Aetas were not screened during the study. Yaws was regarded as a common skin disorder among the Aetas for several years. The psychosocial and economic effects of yaws among the Aetas has recently been reported by Dofitas et al.^13^ Indigenous peoples may actually be missed by local health surveillance due to their semi-nomadic nature and distance from the town centers, enabling diseases like yaws to spread and to remain undetected and untreated. It is likely that other indigenous people (IP) groups in the country have been harboring yaws but have also been missed by local health services.

There is a possibility of past or latent syphilis infection among adults and older adolescents among the Aetas; thus, polymerase chain reaction (PCR) would be required to distinguish between yaws and syphilis. However, this diagnostic test for yaws is not available in our country. Latent or past yaws among this group of Aetas is more likely because of the current and confirmed presence of yaws in their village and because the rural health physician did not know of any report of syphilis in Tagkawayan during his years of work there (Dr K Matundan, personal communication, September 26, 2022). These indigenous people living in the mountains have a low-risk profile for sexually transmitted infections.

Yaws has been found to be more prevalent among indigenous peoples in the African region, but finding yaws cases and treating them is challenging in nomadic populations, such as the Pygmy people in the Central African Republic, Cameroon, Republic of the Congo, and the Democratic Republic of the Congo.^14^ In 2021, a 5-year-old boy of aboriginal descent from the Batek tribe in Malaysia was reported to have active yaws manifesting as yellow-crusted ulcers and granulomatous plaques on the buttocks and thighs. This was the first report of confirmed yaws in Malaysia since 1989, indicating that yaws had been subsequently forgotten by the health services,^15^ as was experienced in the Philippines after yaws ceased to be a notifiable disease. In Malaysia, yaws had existed undetected among indigenous people just like our first yaws cases in Luzon.

The Philippines has 169 living ethnolinguistic groups, with around 140 acknowledged indigenous peoples. Indigenous peoples are present in around 65 out of 81 provinces that make up the Philippines and represent 10–15% of the total Philippine population. The majority of IPs are found in the Mindanao Island group (61%), followed by Luzon (33%) and the Visayas (6%).^16^ Despite the significant number of IPs in the country, there is a dearth of studies on yaws among them. We conducted an electronic search in PubMed, Google Scholar, and DOST SciNet-Phil for reports on yaws among Filipino IPs. The search only yielded one report by Lopez-Rizal et al.^17^ in 1926 about 33 Ifugaos of Northern Luzon who had yaws lesions on muco-cutaneous junctions, such as the mouth and nose (*N* = 22), the anus, and genitalia (*N* = 29), and only five patients also had yaws lesions in areas remote from the mucocutaneous sites. Lopez-Rizal et al.^17^ hypothesized that these “modifications” in the yaws lesions may have been influenced by the altitude and temperature of the mountains where the Ifugaos reside. “Gang-a-gang” was the specific term for yaws among the Ifugaos.^17^

### Active and latent yaws among adults.

Latent yaws cases may be a reservoir of infection because reactivation of yaws may occur over the years. If active and latent yaws cases are combined as sources of infection in the Aeta community, the proportion affected is 20% (7 of 35 screened). Latent yaws cases are often not household contacts of active yaws cases and may be missed if only total targeted treatment is done.^18^ Active yaws among children tends to receive most of the attention in yaws case detection efforts; however, the detection and treatment of latent yaws cases in all ages are also important in achieving eradication of this infection.

The current study also detected the first Filipino adult (25-year-old male) with active yaws, an occurrence that is uncommon because yaws affects children in around 75–80% of the cases, which means 20–25% of cases are 16 years and older.^1^ Initial infections have been observed after the age of 18 years in 15% of cases.^19^ There were only adults with latent yaws and past yaws found in the Mindanao study sites in 2017.^4^ Active yaws among adults may occur especially when the community has had no health services for a long period of time, especially access to antibiotics. Adults may also become infected by children with active yaws in such a cluster of Aeta families.

Historically, yaws has been reported among adults in aboriginal populations in other endemic countries. In 1966, Guthe and Luger^19^ conducted a clinical and serological survey of aborigines in the Northern Territories of Australia and found positive *T. pallidum* immobilization reactions in the sera from 36 (5.6%) of 643 children, compared with 318 (35.4%) of 899 adults. Garner et al.^20^ reported clinical yaws among six aborigines in the Mirrnatja area, North Australia, and reactive treponemal serology among 22 out of the 35 persons tested (62.9%) in this isolated group. In 2021, the WHO reported 15 countries currently endemic for yaws and 76 previously endemic countries, areas, and territories with an unknown yaws status. The Western Pacific Region reported more yaws suspects in Papua New Guinea and the Solomon Islands.^1^

### Yaws bone and joint disease.

Another new finding in the current study is a case of an 8-year-old boy with active yaws skin lesions and bony deformities of the forearms that developed over a few months. This is a case of yaws osteitis or osteoperiostitis affecting the radius and ulnar bones. Radiographs were planned to further confirm and document the skeletal deformities; however, the increasing number of COVID-19 cases in the Tagkawayan municipality did not make it safe for the boy to be brought to the medical facility. This is an important finding because the search for yaws tends to focus on the cutaneous signs rather than the extracutaneous bone signs and symptoms. In the first yaws study conducted in the Mindanao Island group, Southern Philippines, in 2017, one adult respondent recalled having bone pains.^13^

A cross-sectional survey of yaws cases seen in a health facility in Papua New Guinea (*N* = 138) had 92% of participants with osteoarticular involvement, such as arthralgias 48/63 (76.2%) of the large joints (knees, ankles, elbows, and wrists) and bone swelling or marked pain in 10/63 (15.9%).^21^ Mitja et al.^22^ also reported a case series of seven children in Lihir, Papua New Guinea. All cases were diagnosed within 3 weeks to 3 months after the primary yaws lesion appeared. Initial signs and symptoms were bone pain in all cases, soft tissue swelling, and multiple bone involvement. The radial bone was the most commonly affected. Patients with dactylitis had spindle-shaped soft-tissue swelling around the phalanges.

Yaws tends to affect multiple bones and joints (polyostotic) and tends to be bilateral, compared with syphilis, which usually affects a few bones or joints (pauciostotic) and rarely affects the hands or feet. Mitja et al.^22^ reported that the mean number of affected bones in yaws was 3.3 (range: 1–7). Spontaneous healing may occur within a few months. However, if left untreated, some patients can develop destructive bone lesions, such as bowing of the tibia (saber shin), nasal cartilage destruction (gangosa), or exostosis of the paranasal maxilla (gondou), in late yaws. Radiographic signs would show cortical rarefaction and periosteal deposits going on to bony expansion. Osteoperiostitis was successfully treated with benzathine penicillin.^22^ Gonzalez-Beiras et al.^23^ reported one case of osteoperiosteitis of the wrist that was successfully treated with one dose of 500 mg azithromycin in a 5-year-old boy.

### Lessons learned and the next steps.

The important lessons learned from this study are 1) In countries where yaws is not a notifiable disease, yaws is a forgotten disease. 2) To maximize limited research resources, focus case detection efforts on remote, impoverished villages and indigenous peoples in these areas. Even with selective sampling of high-risk communities, yaws can be detected. 3) Include adult members of indigenous people when screening for yaws. Active and latent yaws may be found among adults and can be sources of persisting infection in a village. 4) When screening for yaws, one must also ask about bone pains, swelling, and deformities because these may be common rather than rare. 5) Active surveillance, field research, and community screening for yaws may be limited by COVID-19 pandemic restrictions, but these are still possible with the help of telehealth technology.

What are the next steps? Because there is no yaws program yet in the Philippines, the local government units of endemic communities have to carry on by continuing the mapping of yaws cases among the Aetas of Quezon province and the adjacent Camarines Norte villages, followed by total community treatment and follow-up targeted treatments. Active yaws surveillance was initiated during this study and should be maintained by the participating local health units. Yaws will cease to be a forgotten disease through widespread information campaigns about the clinical appearance of yaws and how it can be confirmed through serological tests. The search for yaws may be integrated with other local Skin Neglected Tropical Diseases programs, such as those for leprosy. More indigenous peoples in isolated villages of the country should be screened for skin diseases and yaws. This will be feasible if government agencies safeguarding the welfare of indigenous peoples also prioritize and expedite health research in order for health policies and services to improve. The WHO has to continue advocating for yaws to be included in the list of notifiable diseases in all countries because yaws is targeted for eradication.^2^

## LIMITATIONS

The following events and factors led to limitations in this study and resulted in the underreporting of yaws cases especially in Tagkawayan, Quezon, and Rodriguez, Rizal, where IPs were screened. This study had limited study sites and number of participants owing to budgetary constraints. We could not perform PCR confirmation of the etiologic agent of active yaws skin lesions due to the lack of PCR facilities within the Philippines and a budget to send specimens to laboratories abroad. The funds and diagnostic capacity for PCR confirmation of yaws are generally lacking, as reported by Handley et al.^24^ in a recent survey of yaws-endemic countries, including the Philippines. Ideally, PCR to confirm *T. pallidum pertenue* would have to be performed on yaws-like skin ulcers among children because concomitant infection caused by *Hemophilus ducreyii* is also possible.^25^ Despite this limitation, we did confirm the presence of treponemal antibodies in all yaws cases with skin ulcers using the RDT and DPP.

The COVID-19 pandemic caused major disruptions in the implementation of the study because community quarantines and COVID-19 cases kept health services busy and prevented field work in most parts of the country starting March 2020. The focus of healthcare workers on the COVID-19 problem may have led to a low response rate of the active surveillance efforts of the investigators and, thus, underreporting of yaws suspects. Our field work was also delayed by natural disasters such as the Taal Volcano eruption in January 2020 and lengthy NCIP clearance processes.

## CONCLUSION

We detected the first cases of yaws among the Aetas, an indigenous people of Quezon Province in the Luzon Island group. Two major island groups, Luzon and Mindanao, now have confirmed yaws cases, but there were no yaws cases detected in selected villages of the Visayas Island group. The burden of yaws in the Philippines is still underestimated due to the low awareness level among Filipinos, the very limited sampling of epidemiological studies, and the absence of a nationwide active surveillance for yaws.

## Supplemental files


Supplemental materials
