# Supplementary material for: Finding Yaws among Indigenous People: Lessons from Case Detection Surveys in Luzon and Visayas Island Groups of the Philippines
Source: Am J Trop Med Hyg. 2022 Dec 26;108(2):305–12. doi: 10.4269/ajtmh.22-0566 (PMC9896346; doi:10.4269/ajtmh.22-0566)
Supplement: Supplementary file 1 [file tpmd220566.SD1.pdf]

S1. WHO-recommended clinical definitions of yaws cases

Rumour of yaws: A person identified by a community member or volunteer with suspicion of yaws.

Suspected yaws case: A person of any age who is or was living in a previously or currently endemic area and who presents with clinical signs consistent with yaws.

Treponemal positive case: A case of suspected yaws with a positive rapid treponemal point-of care test (i.e. both treponemal and control lines visible) or a positive *T. pallidum* haemagglutination assay or *T. pallidum* particle agglutination assay.

Serologically confirmed yaws case: A suspected case confirmed by dual positive serology: in a dual path platform (treponemal and non-treponemal) (DPP) test or a *T. pallidum* haemagglutination or *T. pallidum* particle agglutination assay plus rapid plasma reagin test.

PCR-confirmed yaws case: A case that is positive in a polymerase chain reaction (PCR).

Other WHO-recommended definitions

People with past or current yaws infection

Person with past yaws infection: Someone with DPP results showing a reactive treponemal test and a non-reactive non-treponemal line; may be asymptomatic or symptomatic.

Person with active yaws infection: Someone with positive dual serological tests; may be asymptomatic or symptomatic (the latter being a serologically confirmed yaws case).

Case of infectious yaws: A clinical case (suspected, treponemal positive or confirmed) of yaws that was not treated and could potentially infect other people.

Person with latent yaws infection: Someone with positive DPP results but no current yaws-like clinical symptoms. Among cases that were not treated but healed spontaneously, the disease may enter a latent phase with no lesions."

Reference: Eradication of yaws: surveillance, monitoring and evaluation. A manual for yaws eradication programme managers. Geneva: World Health Organization; 2021. Licence: CC BY-NC-SA 3.0 IGO. Page viii.

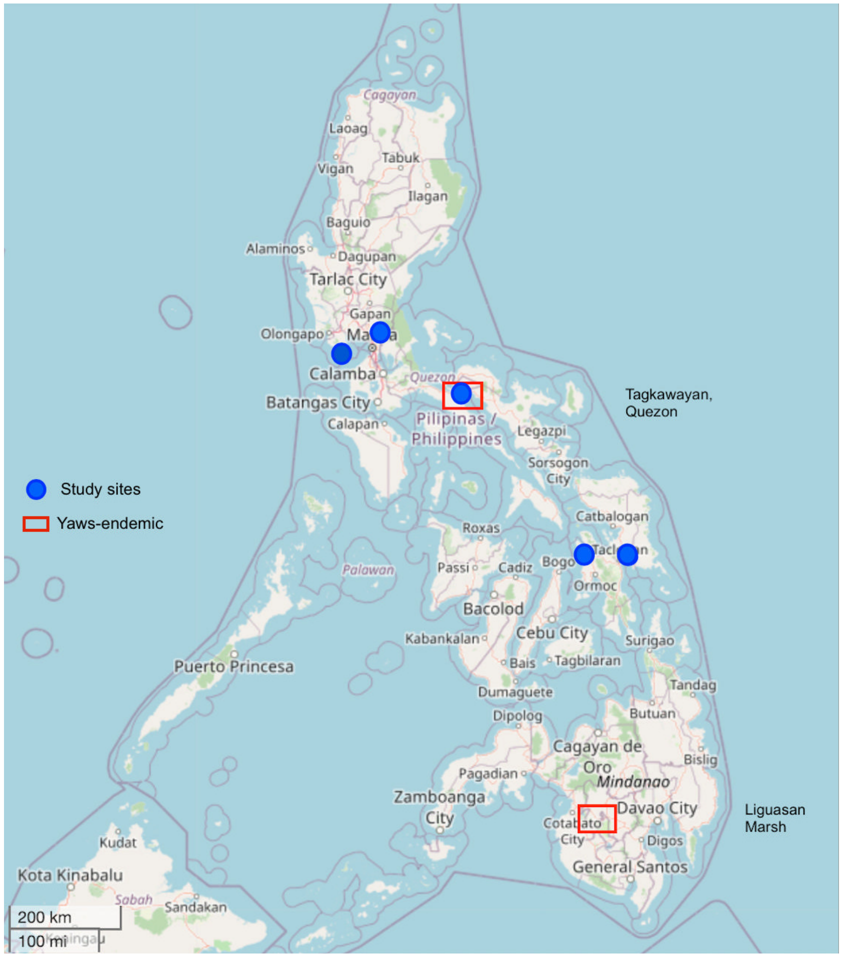

S2. Location map of study sites & yaws-endemic areas

(Contains information from OpenStreetMap and OpenStreetMap Foundation, which is made available under the Open Database License)

S3. CLINICAL & SEROLOGICAL DETAILS OF YAWS CASES
(N = 19)

No.	INITIALS	AGE	SEX	DPP RESULT<br>1<br>TREPONEMAL ANTIBODY	2<br>NON-TREPONEMAL ANTIBODY	3<br>CONTROL	INTERPRETATION	CLINICAL DIAGNOSIS	Patient Group	History & Skin findings
**ACTIVE YAWS n = 5**										
1	RA	8	M	REACTIVE	REACTIVE	POSITIVE	Active & Untreated	Secondary Yaws with bony deformities (forearms)	Child	1 month history - Small ulcers & ulcerated nodules on dorsum of feet, ankle area, and dry nodules on legs; with bleeding & pain; forearms - bowing deformity of few months duration
2	LP	10	F	REACTIVE	REACTIVE	POSITIVE	Active & Untreated	Secondary Yaws; Plantar yaws	Child	Large thick brown-yellow crusted clustered nodules on R lower leg below knee; solitary dry yellow-crusted nodule on base of 1st toe L foot & solitary grey-black round nodule adjacent; the right foot had a healing ulcerated nodule on the medial aspect. Both soles of the feet had healing or healed erosions and a small erosion on the lateral aspect of the L sole
3	RP	11	M	REACTIVE	REACTIVE	POSITIVE	Active & Untreated	Secondary Yaws; Plantar yaws	Child	Yellow- crusted small ulcers on the dorsum of the right foot and dry scaly round nodules on the 2nd and 3rd toes; multiple atrophic round scars and black crusts around the ankles; multiple small erosions and curvilinear wounds on the right sole

										and 1st and 2nd toes
4	JP	7	F	REACTIVE	REACTIVE	POSITIVE	Active & Untreated	Secondary Yaws; Plantar Yaws; pitted keratolysis	Child	Large, dry, skin-colored and whitish, papulosquamous plaques on the dorsum of her left foot extending to her ankle and medial aspect of the soles and a similar plaque on her right heel and ankle. There was a large round dry crusted nodule on the dorsum of her right foot and a large round erosion on the base of her first toe ; large irregular erosions on the R sole especially the heel and on the first toe L foot, with small pits on the L sole on the heel
5	RA	25	M	REACTIVE	REACTIVE	POSITIVE	Active & Untreated	Secondary Yaws; Plantar Yaws	Adult	Multiple, red-crusted ulcerated nodules on anterior L leg; Multiple, small round yellow-crusted ulcers on heel R foot; L foot: multiple large dry erosions on sole and toes; multiple red-brown crusted nodules with peripheral scaling lateral aspect of L foot; R sole and toes - one large brownish papilloma with peripheral scaling; multiple small yellow crusted papules; multiple small and large erosions ; moved to Camarines Norte
**LATENT YAWS n = 2**										
6	CA	30	F	REACTIVE	REACTIVE	POSITIVE	Active & Untreated	Latent Yaws; Tinea imbricata	Adult	Pruritic, whitish macules and patches on nape, legs, feet 3 months duration; tinea imbricata (2019 treated); has HH contact with past yaws

7	RA	24	M	REACTIVE	REACTIVE	POSITIVE	Active & Untreated	Latent Yaws	Adult	Multiple, round, well-defined scars on dorsum of both feet; one year duration papule on left foot; similar skin lesions in wife (did not consent to skin exam); has HH contact with past yaws
**PAST YAWS n = 12**										
8	JA	35	M	REACTIVE	NON-REACTIVE	POSITIVE	Past/treated	Past yaws; Plantar yaws vs pitted keratolysis; Tinea imbricata	Adult	2-3 years history- multiple wounds on both feet; scaly macules and hypertrophic scars with pruritus, pain; numerous small erosions on plantar aspect of toes, large erosion on sole and heels; also with tinea imbricata
9	MV	16	F	REACTIVE	NON-REACTIVE	POSITIVE	Past/treated	Past yaws	Child	L arm: solitary dark brown ulcerated and crusted nodule peri-axillary; multiple atrophic scars on knees; dorsa of feet - multiple small round erosions with peripheral dark brown pigmentation and scaling
10	RA	25	M	REACTIVE	NON-REACTIVE	POSITIVE	Past/treated	Past yaws; tinea imbricata	HH contact of Latent yaws	2 years history- wound on right foot; multiple whitish scaly macules and hypertrophic scars on dorsum of feet; similar lesions in wife (did not consent to skin exam); also with concentric scaly plaques on arms (tinea imbricata)
11	RA	23	M	REACTIVE	NON-REACTIVE	POSITIVE	Past/treated	Past yaws; Pityriasis versicolor	Adult	Several years duration of wounds on dorsum of right foot and legs; PE: multiple white follicular scaly macules on arms, trunk, legs P versicolor), white scaly patches & hypertrophic scars on foot; no other household members with similar lesions; also with onychomycosis and infected wound; HH contact negative serology

12	GA	22	F	REACTIVE	NON-REACTIVE	POSITIVE	Past/treated	Past yaws	Adult	Several years history of wound on dorsum of 2nd digit left foot, both legs and feet; associated pruritus and pain/tenderness; applied diesel; husband with scars on legs; scaly macules on legs and dorsa of feet; hypertrophic scar on 2nd toe
13	AA	44	F	REACTIVE	NON-REACTIVE	POSITIVE	Past/treated	Past yaws	Adult	Several years history of multiple wounds/ulcers on lower legs ; associated pain; applied diesel; multiple atrophic scars on lower legs
14	DJ	52	F	REACTIVE	NON-REACTIVE	POSITIVE	Past/treated	Past yaws	Adult	8 months history- scaling macules and ulceration on left ankle, both feet; initially pruritic, painful; applied diesel; other household members with similar lesions; PE: atrophic scars
15	JA	22	M	REACTIVE	NON-REACTIVE	POSITIVE	Past/treated	Past yaws	HH contact active yaws	2 years history- ulcerating wound on lower extremities; pruritus and pain; applied diesel; other household members with similar lesions; atrophic scars on knees; hypertrophic scar on dorsum of right foot; abrasion on R lower leg 1 week duration
16	NA	50	M	REACTIVE	NON-REACTIVE	POSITIVE	Past/treated	Past yaws	HH contact Latent yaws	2 years of recurrent wounds on feet, pruritic and tender; whitish macules and scars developed; multiple hypertrophic scars on dorsa of feet; spouse of past yaws case
17	NA	50	F	REACTIVE	NON-REACTIVE	POSITIVE	Past/treated	Past yaws	HH contact Latent yaws	3 years of recurrent wounds on both feet and legs; treated with diesel; husband with hypertrophic scars; adult daughter with macules; spouse of past yaws case

18	RM	31	M	REACTIVE	NON-REACTIVE	POSITIVE	Past/treated	Past yaws	Adult	Several years history- papules on dorsal aspect of right foot; had ulcers on ankle flexure; multiple scars on both feet; associated pain; wife has scars on both feet, papules on arm; hypertrophic scars on dorsum of R foot and ankle flexures; Spouse of past yaws case
19	JM	30	F	REACTIVE	NON-REACTIVE	POSITIVE	Past/treated	Past yaws	Adult	Several years history - papule on 4th digit left foot, left arm; developed other papules that ulcerated on both feet; associated pain; papule on L arm; husband with multiple scars on feet; hypertrophic scars on dorsum of foot; solitary small eroded brown papule on L forearm; Spouse of past yaws case

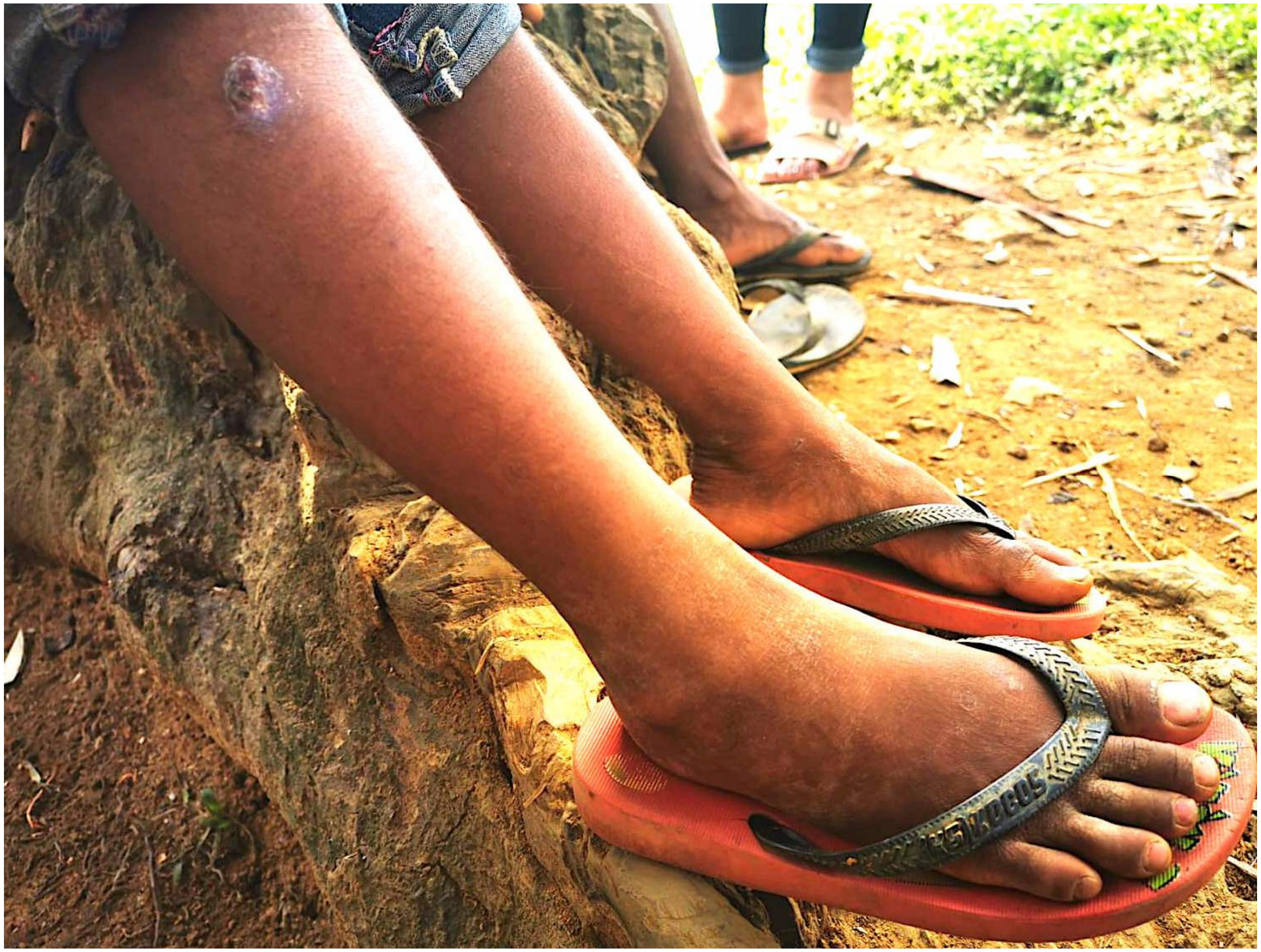

S4. Case 2. A 10-year-old female had large, thick brown-yellow crusted nodules on the right lower leg just below the knee. There was also a solitary dry, yellow-crusted nodule on the base of the first toe on the left foot and a solitary grey-black round nodule adjacent to it. The right foot had a healing ulcerated nodule on the medial aspect. Both soles of the feet bore signs of plantar yaws that were healing and a small erosion on the lateral aspect of the left sole.

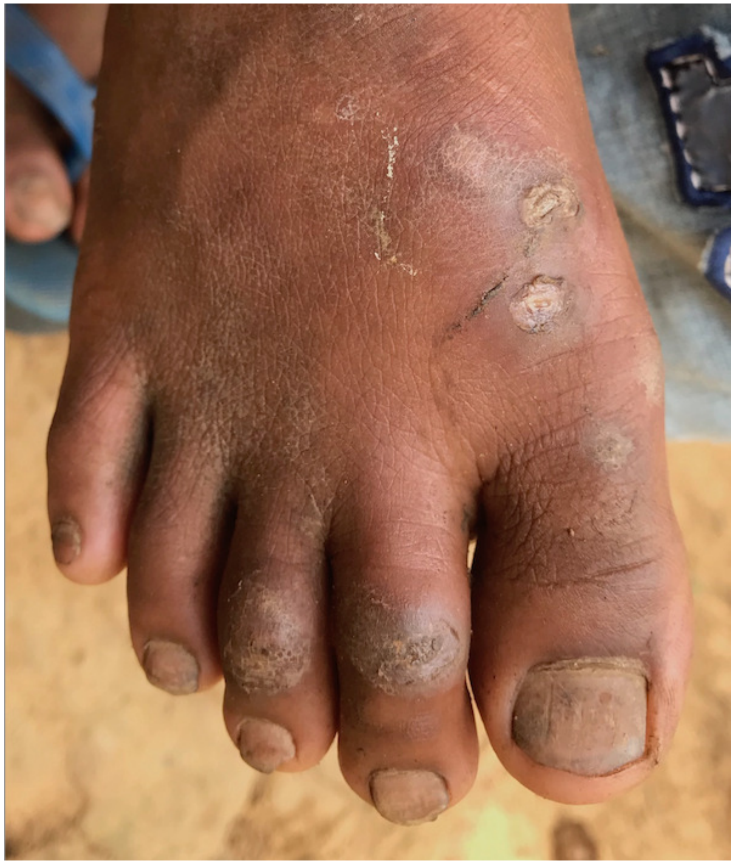

S5. Case 3. An 11-year-old male had yellow- crusted small ulcers and round nodules on the on the dorsum of the right foot, and multiple atrophic round scars and black crusted erosions around the ankles. (S Figure 8) He had plantar yaws: multiple small erosions on the right sole and first and second toes.

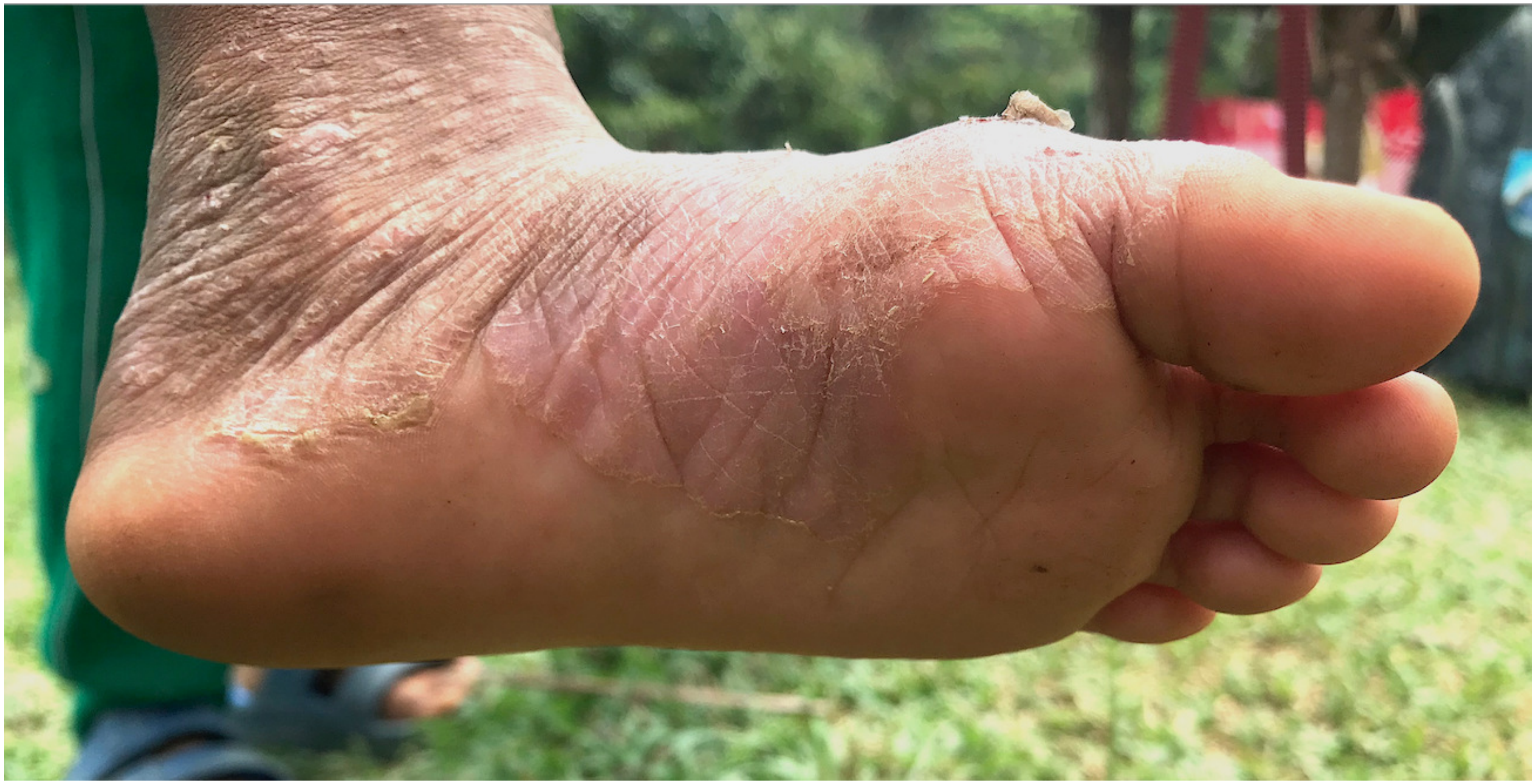

S6. Case 4. A 7-year-old female had large, dry, skin-colored and whitish, papulosquamous plaques on the dorsa of her feet , ankles, and soles. There was a large, round, dry, crusted nodule on the dorsum of her right foot and a large round erosion on the base of her first toe. (Photo credit. Dr Belen Dofitas)

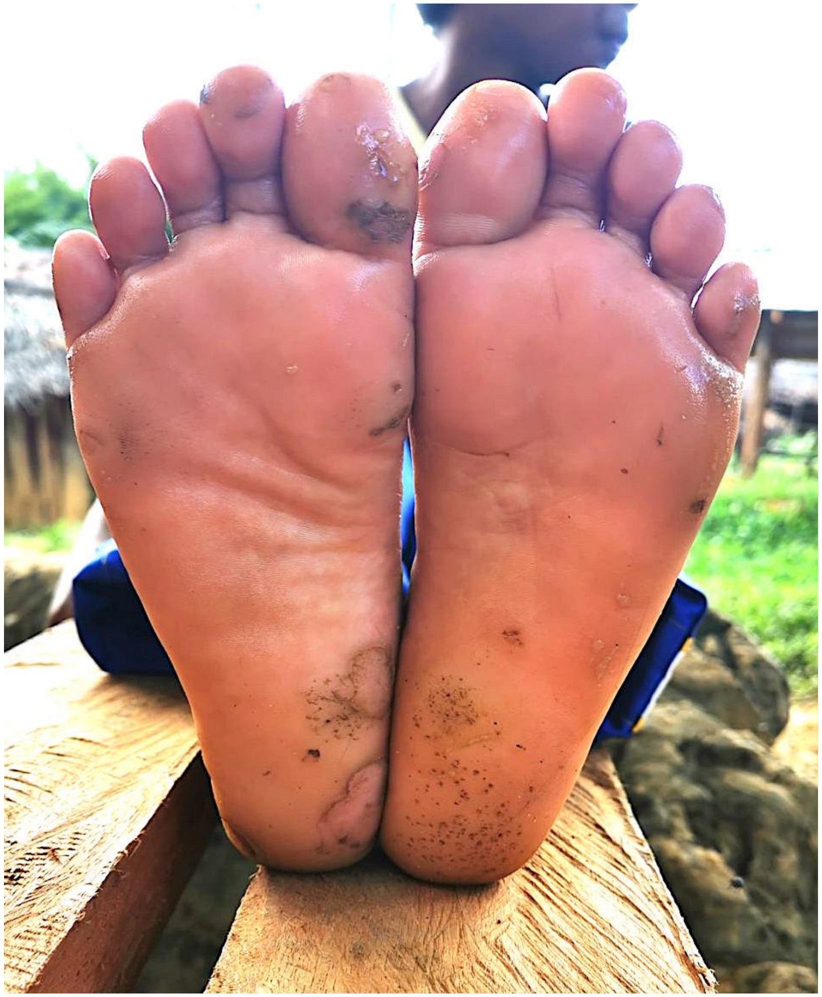

S7. Case 4: Plantar yaws: large irregular erosions on the right sole, especially the heel, and on the first toe of the left foot, with small pits on the left sole on the heel (pitted keratolysis) (Photo credit: Dr Kevin Matundan)

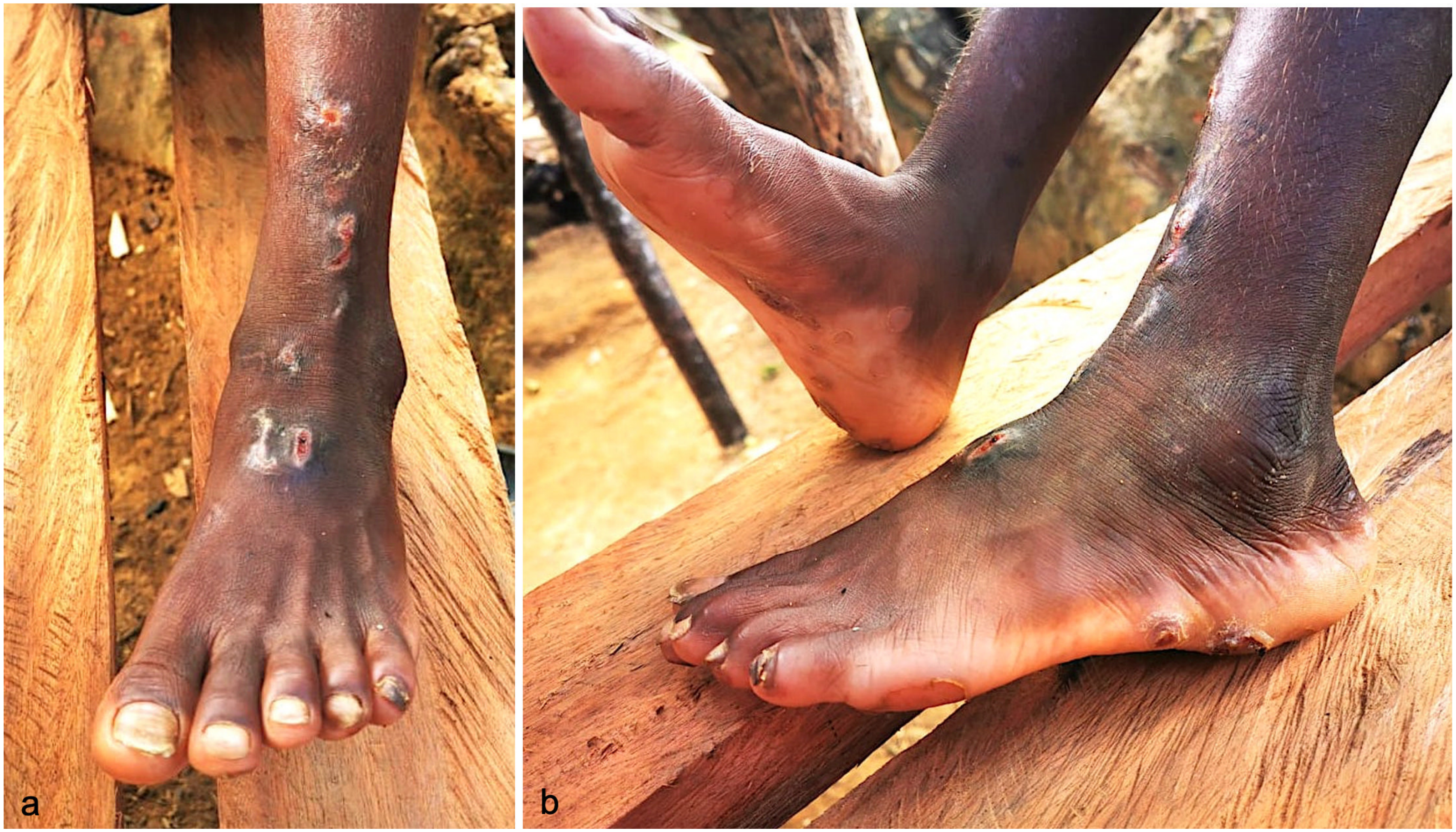

S8. Case 5: Active yaws in an adult Aeta: A 25-year-old male had multiple, red-crusted, ulcerated nodules on the anterior left leg and multiple, small round, yellow-crusted ulcers on heel of the right foot. (Photo credits: Dr. Kevin Matundan)

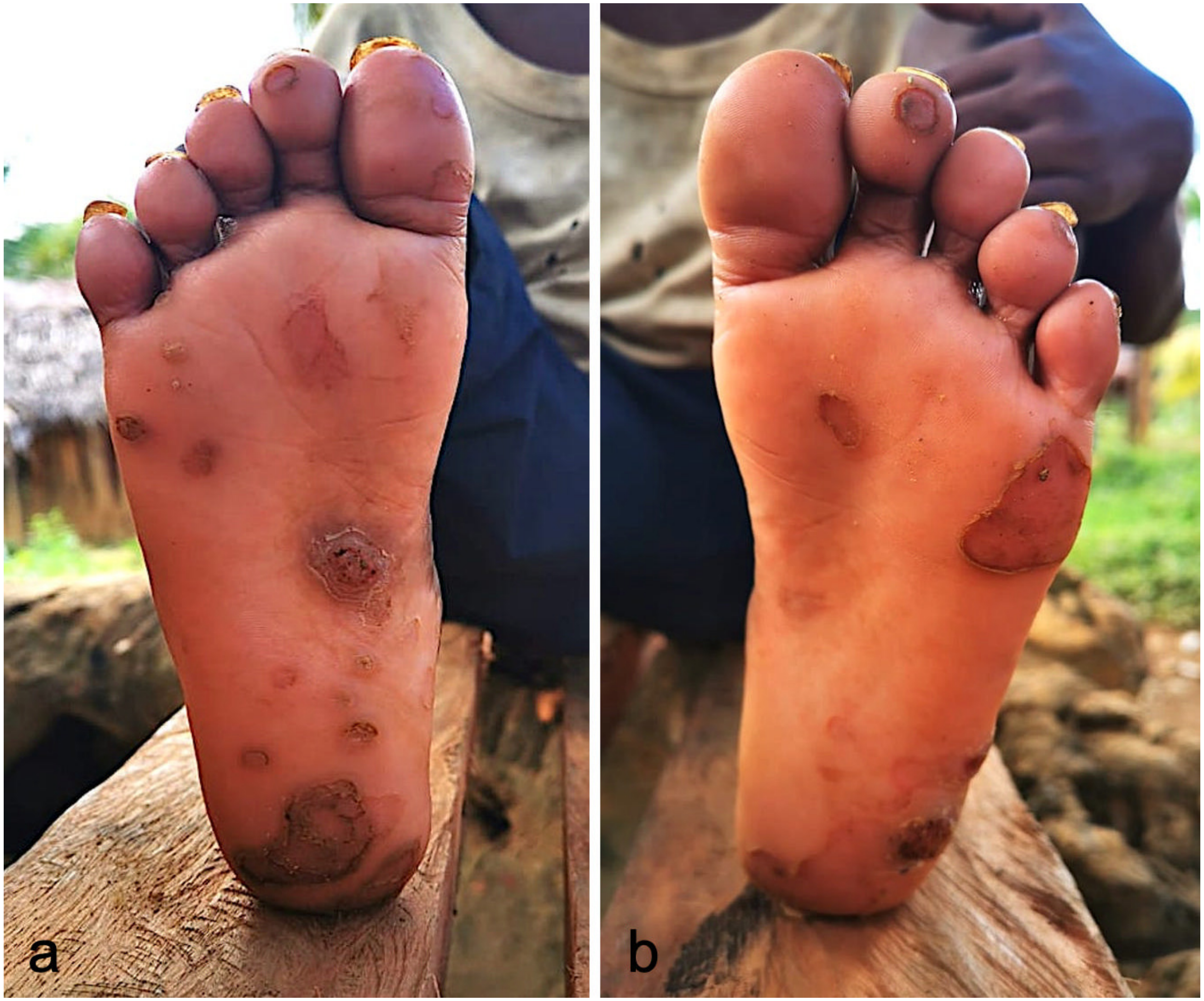

S9. Case 5: Plantar yaws on the soles and toes - small and large brownish papillomas with peripheral scaling; multiple, small yellow crusted papules; multiple small and large erosions (Photo credits: Dr. Kevin Matundan)

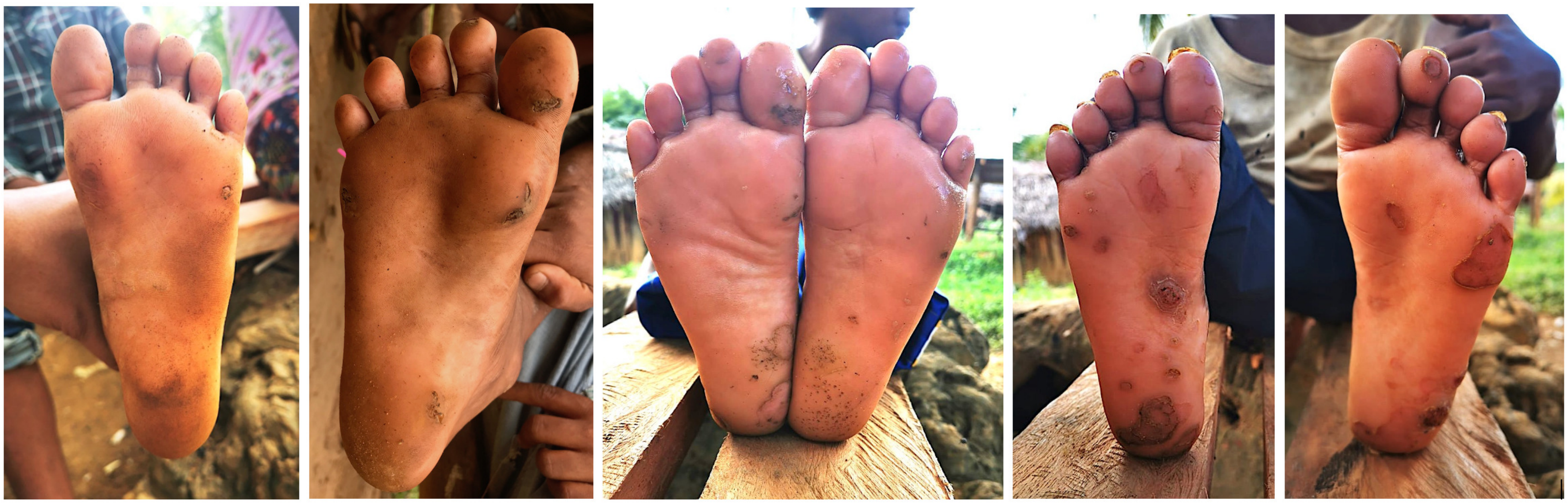

S10. Clinical features of plantar yaws cases among Aetas

Case 2. 10/F Healing plantar yaws: round, superficial erosions with peripheral scaling and with central, dark brown, round patch corresponding to the resolved papilloma; round erosions - medial; small oval erosion - lateral

Case 3. 11/M Curvilinear erosions - medial, heel; irregular small ulcers- toe, lateral sole; punched out ulcer - toe

Case 4. 7/F. R foot: Large, oval, and round erosions on the heel; curvilinear erosion - medial; irregular healing erosions - toe, lateral; punched out, dry ulcer - R lateral heel; pitted keratolyisis - both heels

Case 5. 25/M Yaw papilloma with peripheral scale - R mid-sole & L heel; healing yaw papilloma - R heel; small papilloma - L lateral sole; multiple, small, punched out ulcers - toes; large and deep erosions L mid-sole & lateral, both heels

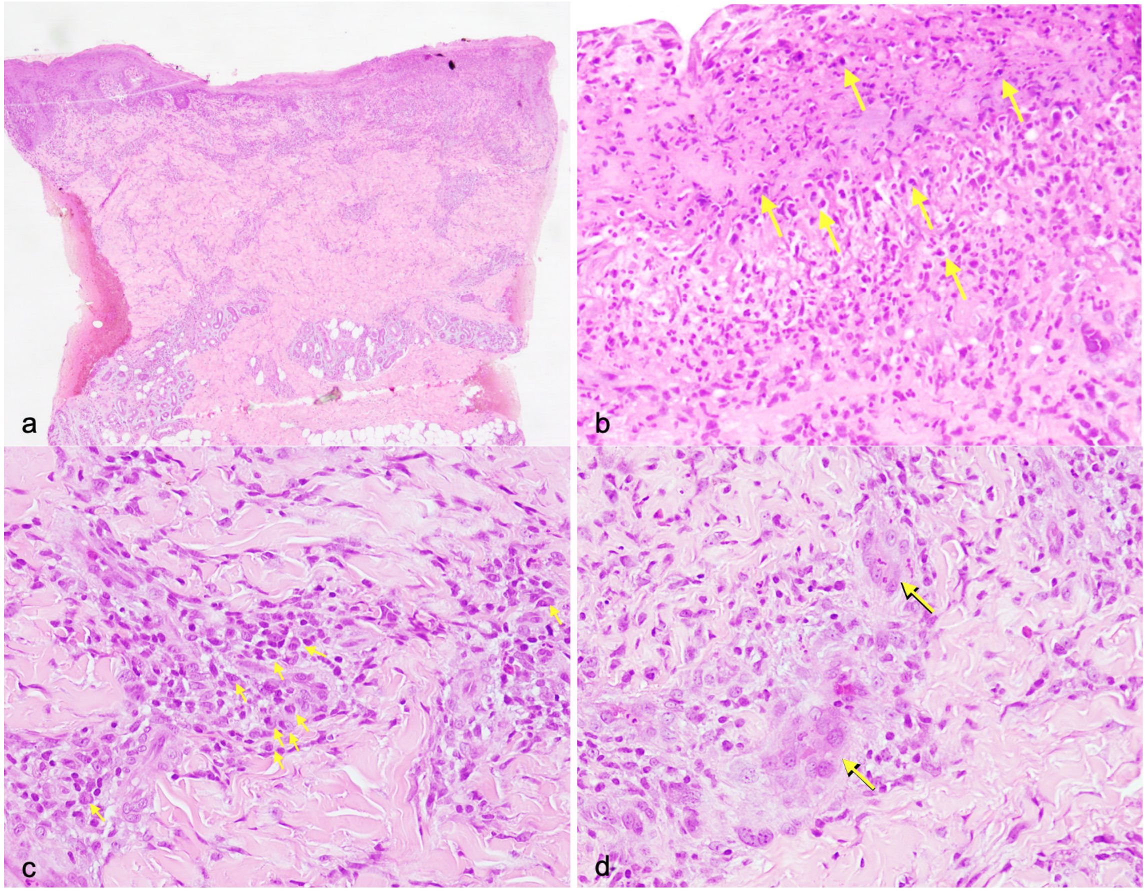

S11. Histopathological findings of a yaws skin ulcer (Case 1): a) Psoriasiform epidermal hyperplasia and complete epidermal thickness loss; dense, bandlike infiltrate of lymphoyctes, neutrophils and plasma cells in the papillary dermis and arrayed perivascularly in the reticular dermis; subcutaneous septal thickening is present (Scanning view); b) Overlying scale crust of neutrophils and plasma cells; exocytosis of neutrophils in the epidermis (High power view); c) Plasma cells in the papillary dermis and arrayed perivascularly in the reticular dermis (High power view); d) Endothelial swelling of blood vessels (High power view) (Photo credits: Dr Len C Balmores).
